# Embryonic Development of the Deer Mouse, *Peromyscus maniculatus*

**DOI:** 10.1371/journal.pone.0150598

**Published:** 2016-03-01

**Authors:** Shannon W. Davis, Jessica L. Keisler

**Affiliations:** Department of Biological Sciences, University of South Carolina, Columbia, SC, United States of America; New York Medical College, UNITED STATES

## Abstract

Deer mice, or *Peromyscus maniculatus*, are an emerging model system for use in biomedicine. *P*. *maniculatus are* similar in appearance to laboratory mice, *Mus musculus*, but are more closely related to hamsters than to *Mus*. The laboratory strains of *Peromyscus* have captured a high degree of the genetic variability observed in wild populations, and are more similar to the genetic variability observed in humans than are laboratory strains of *Mus*. The *Peromyscus* Genetic Stock Center at the University of South Carolina maintains several lines of *Peromyscus* harboring mutations that result in developmental defects. We present here a description of *P*. *maniculatus* development from gastrulation to late gestation to serve as a guide for researchers interested in pursuing developmental questions in *Peromyscus*.

## Introduction

Members of the genus *Peromyscus* are widely distributed throughout North America, and are represented by several species of mice, including deer mice (*P*. *maniculatus*), white footed mice (*P*. *leucopus*), and oldfield mice (*P*. *polionotus*). While superficially resembling *Mus musculus*, the common laboratory mouse, *Peromyscus* are more closely related to hamsters than to *Mus* or *Rattus* (rats), having shared a last common ancestor with *Mus* and *Rattus* approximately 25 million years ago [1]. *Peromyscus* have many similar characteristics as *Mus*, including a small size (approximately 25 g) and a short generation time for mammals, which makes them a useful model system for laboratory studies [2]. *Peromyscus* typically live four to five years in captivity, and remain reproductive for at least two years [3]. Additionally, laboratory strains of *Peromyscus* have maintained the genetic variability of wild populations and are, therefore, more similar to the genetic variability observed in humans than *Mus* [4]. Extensive reviews of *Peromyscus* as a model system expands on these and other topics and serve as an excellent introduction to *Peromyscus* research [3,5]. The *Peromyscus* Genetic Stock Center (PGSC) at the University of South Carolina facilitates the use of *Peromyscus* laboratory research by supplying five species of *Peromyscus* and several lines of *Peromyscus maniculatus* harboring unidentified mutations that cause coat color, neurologic, or developmental defects (http://stkctr.biol.sc.edu). Laboratory strains of three additional species of *Peromyscus* and four subspecies of *P*. *maniculatus* are housed at the University of New Mexico, the University of Illinois Urbana-Champaign, and Harvard University [5].

The natural genetic variation between species and subspecies of *Peromyscus* is a rich genetic resource that has been instrumental in determining sequence variations that result in phenotypic changes in wild populations. Two species of *Peromyscus*, *P*. *maniculatus*, and *P*.*polionotus*, can produce viable, fertile offspring when hybridized. The generation of hybrid offspring has enabled the production of a *Peromyscus* genomic linkage map and the ability to map genetic loci linked to specific behaviors or phenotypes [6,7]. For instance, burrowing behavior differs between the two species such that *P*. *polionotus* dig burrows with longer entrance tunnels and an escape tunnel, while *P*. *maniculatus* dig shorter entrance tunnels and do not dig an escape tunnel [8]. F_1_ hybrids between the two species dig burrows similar to *P*. *polionotus*, indicating that the *P*. *polionotus* burrowing behavior is a dominant phenotype. Quantitative trait locus (QTL) analysis of a first backcross generation of F_1_ hybrids to *P*. *maniculatus* demonstrates that entrance tunnel length is determined by three loci, and the presence of an escape tunnel by a single locus [8]. The natural genetic variation between *Peromyscus* subspecies has been instrumental in identifying single-nucleotide polymorphisms (SNPs) that result in coat color differences between the subspecies. In *P*. *polionotus* a QTL analysis between light colored beach mice and dark colored inland mice identified three QTLs that each contain a candidate gene known to affect coat color in *Mus* [9]. One candidate gene, *melanocortin-1 receptor*, contains a SNP in the coding sequences that results in the lighter coat color of some of the *P*. *polinoutus* subspecies on the Florida Gulf coast [10]. A second candidate gene, *Agouti*, contains a SNP in a *cis*-regulatory sequence that shits the dorsal/ventral boundary of expression of *Agouti* during embryonic development [11]. The increased expression of *Agouti* in more dorsal locations results in a lighter pigmentation of beach dwelling *P*. *polinoutus* [11]. The application of next-generation sequencing technology in *P*. *maniculatus* has also identified 10 SNPs in the *Agouti* locus, which in combination result in the lighter coat color of deer mice found in the Nebraska Sand Hills [12]. The continued identification of *Peromyscus* genomic sequence variants will be aided by the genomic sequencing projects of several *Peromyscus* species. The first assembly of the *P*. *maniculatus* genome is now available, and draft sequences of *P*. *polionotus*, *P*. *leucopus*, and *P*. *californicus* are available (https://www.hgsc.bcm.edu/peromyscus-genome-project).

Some of the *P*. *maniculatus* mutations available at the PGSC, including *cataract-webbed (cw)*, *dominant spot (S)*, and *tan streak (tns)*, potentially harbor developmental defects [3]. *Cataract-webbed* is a homozygous recessive mutation with syndactyly of the middle digits at birth and cataract formation by eight months of age [3,13]. *Dominant spot (S/+)* animals have a white blaze on the forehead, similar to piebaldism in humans, and are likely to be embryonic lethal when homozygous [3,14]. The similarity in phenotype between *dominant spot* in *Peromyscus* and known spotting mutations in humans and *Mus* suggests a neural crest defect. *Tan streak* (*tns/tns*) is characterized by a pigmented stripe running along the dorsal midline and white fur covering the rest of the body [3,15]. This phenotype suggests a failure in melanocyte migration during development, but remains to be verified.

Characterization of these developmental mutations or developmental changes caused by natural variation requires knowledge of the developmental timing of *P*. *maniculatus*. *P*. *maniculatus* gestation is approximately 24 days, compared to 21 days in *Mus*. Manceau et al. presented an initial developmental time line of *P*. *maniculatus* from e10 to e22 [11]. We have extended this initial report by collecting embryos from earlier time points and presenting additional images that follow the development of external structures throughout development. We also present a comparison of *P*. *maniculatus* development to *M*. *musculus* development in order to serve as a guide for investigators pursuing *Peromyscus* developmental biology.

## Materials and Methods

All experiments were approved by the University of South Carolina Institutional Committee on the Use and Care of Animals.

Male and female BW *P*. *maniculatus* (*Peromyscus* Genetic Stock Center, University of South Carolina) were housed together on a 16 to 8 hour light/dark cycle, and feed food and water *ad libitum*. Unlike *M*. *musculus*, *P*. *maniculatus* do not produce a reliably visible copulation plug. A vaginal lavage was performed on females each morning to assay for the presence of sperm. Noon of the day sperm was detected was designated as embryonic day 0.5 (e0.5). Pregnant females were euthanized using CO_2_ asphyxiation on each day of embryonic development between e8.5 and e21.5, and embryos were isolated in 1 x phosphate buffered saline, pH7.4 (PBS). Isolated embryos were fixed in 4% paraformaldehyde in PBS overnight at 4 C. Following fixation, embryos were washed in PBS and then dehydrated through 50% ethanol to 70% ethanol and stored at -20 C. For photography embryos were hydrated through 50% ethanol to PBS. 1% agarose in PBS was melted and poured into standard tissue culture plates and allowed to solidify. The agarose pad was covered with PBS and embryos placed on top of the agarose. Pits dug into the agarose with forceps allowed for positioning of embryos to maintain specific positions for photography. All images were collected using a Leica DFC290 HD camera mounted on a Leica M165FC stereomicroscope, using mainly dark field illumination with additional lighting from above as necessary, and bright field illumination of e8.5 and e9.5 embryos.

## Results

**Fig 1:**
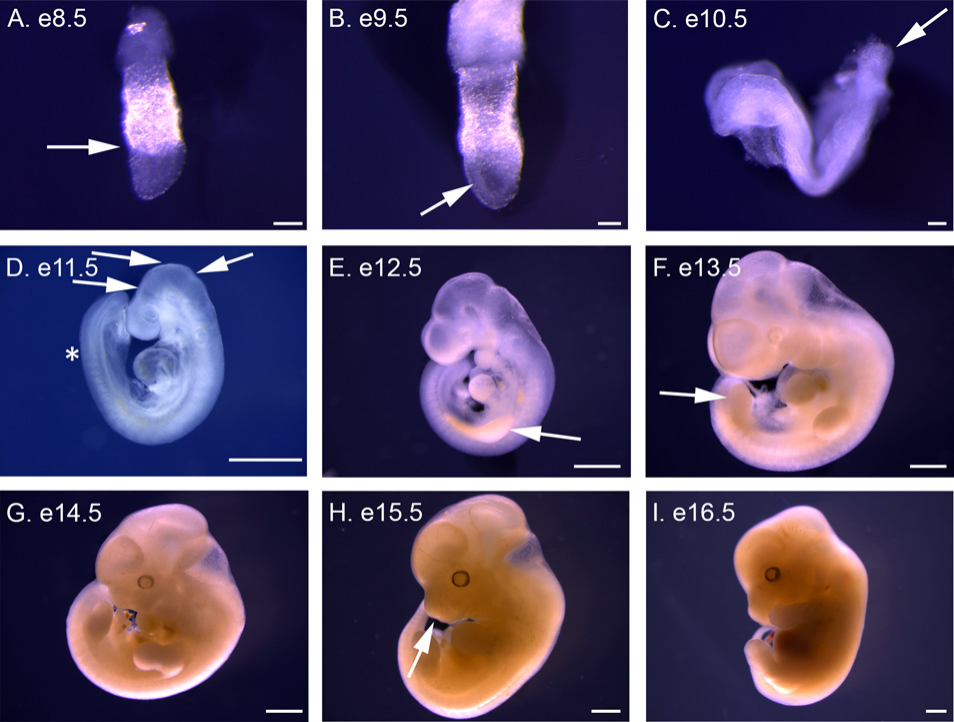
Lateral views of *P*. *maniculatus* embryos from e8.5 to e16.5. (A) e8.5 embryo. Arrow indicates division between extra-embryonic and embryonic tissues and the anterior of the embryo. (B) e9.5 embryo. Arrow indicates thickening neural plate. (C) e10.5 embryo. Arrow indicates the allantois at the posterior of the embryo. (D) e11.5 embryo. Arrows indicate the divisions between the telencephalon and diencephalon, diencephalon and mesencephalon, and mesencephalon and rhombencephalon, moving from left to right. Asterisk indicates the boundary between the presomitic mesoderm and the last somite. (E) e12.5 embryo. Arrow indicates forming forelimb bud. (F) e13.5 embryo. Arrow indicates forming hindlimb bud. (G) e14.5 embryo. (H) e15.5 embryo. Arrow indicates forming nares. (I) e16.5 embryo. Scale bars in A–C equal 100 μm. Scale bars in D–N equal 1 mm.

**Fig 2:**
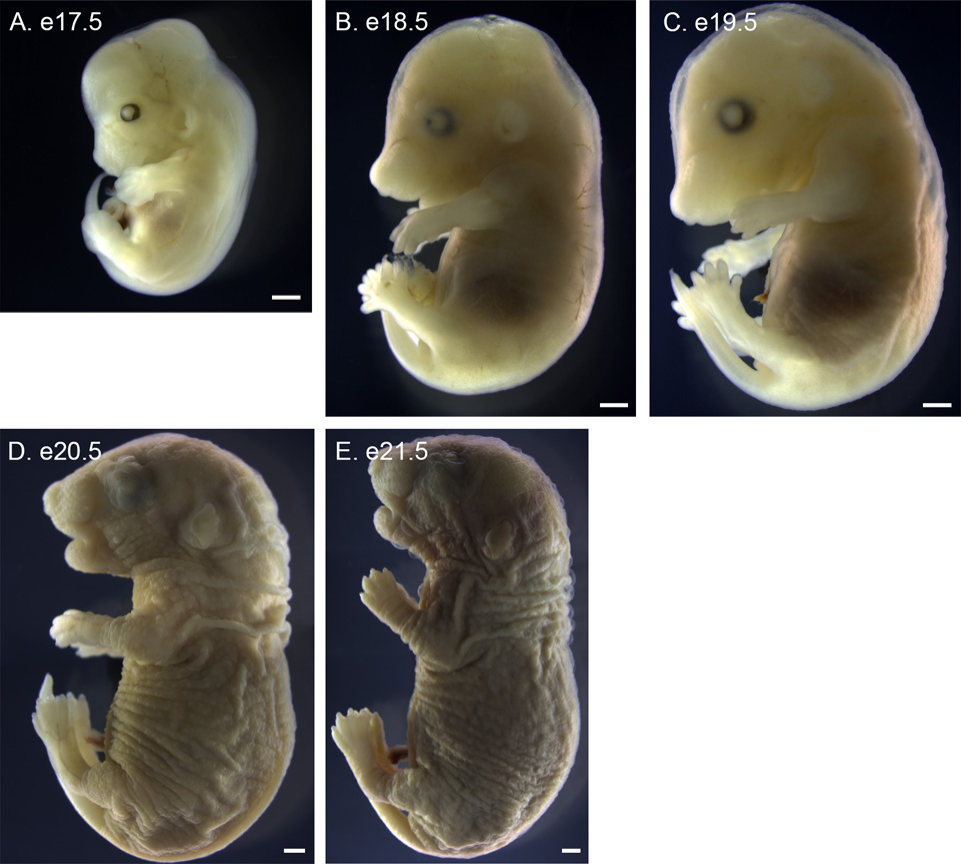
Lateral views of *P*. *maniculatus* embryos from e17.5 to e21.5. (A) e17.5 embryo. (B) e18.5 embryo. (C) e19.5 embryo. (D) e20.5 embryo. (E) e21.5. Panels D and E are each a composite of images overlaid to visualize the entire embryo. All scale bars equal 1 mm.

**Fig 3:**
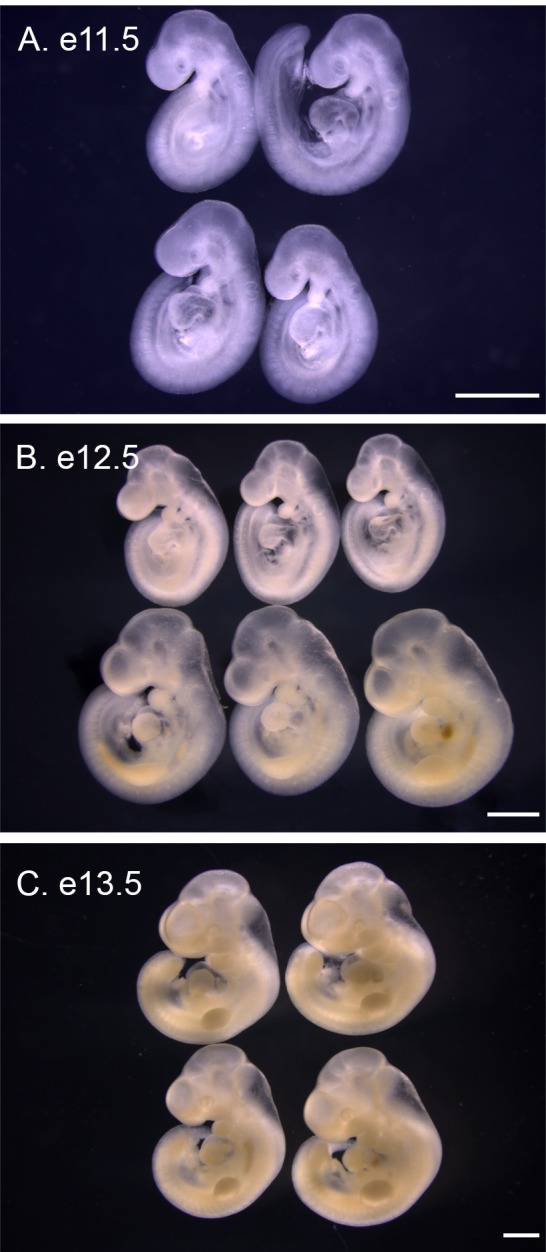
Variability between individuals of the same litter and between litters of *P*. *maniculatus* embryos. (A) One litter of four e11.5 embryos. (B) Two litters of three e12.5 embryos, separated into rows. (C) One litter of four e13.5 embryos. All scale bars equal 1 mm.

*P*. *maniculatus* embryos were collected on each day of development between e8.5 to e21.5 to provide an initial atlas of external embryonic development (Fig 1 and Fig 2). As with *M*. *musculus*, *P*. *maniculatus* displays variability between individual embryos within the same litter and between litters collected on the same day of development (Fig 3). *P*. *maniculatus* have litters with between 3 and 5 embryos, compared to 6 to 8 embryos per litter for inbred *M*. *musculus* strains. Within one litter, size is the common variability between individuals (Fig 3). Developmental timing can vary with between litters collected at the same gestational time (Fig 3B). For this atlas only one embryo for each developmental day is presented, with the understanding that significant variation in both size and developmental timing can occur on each day of gestation. Because of the similarity between *P*. *maniculatus* and *M*. *musculus* development, this survey is not intended to be exhaustive, but rather to highlight key developmental time points that may add future research into specific developmental systems.

**Fig 4:**
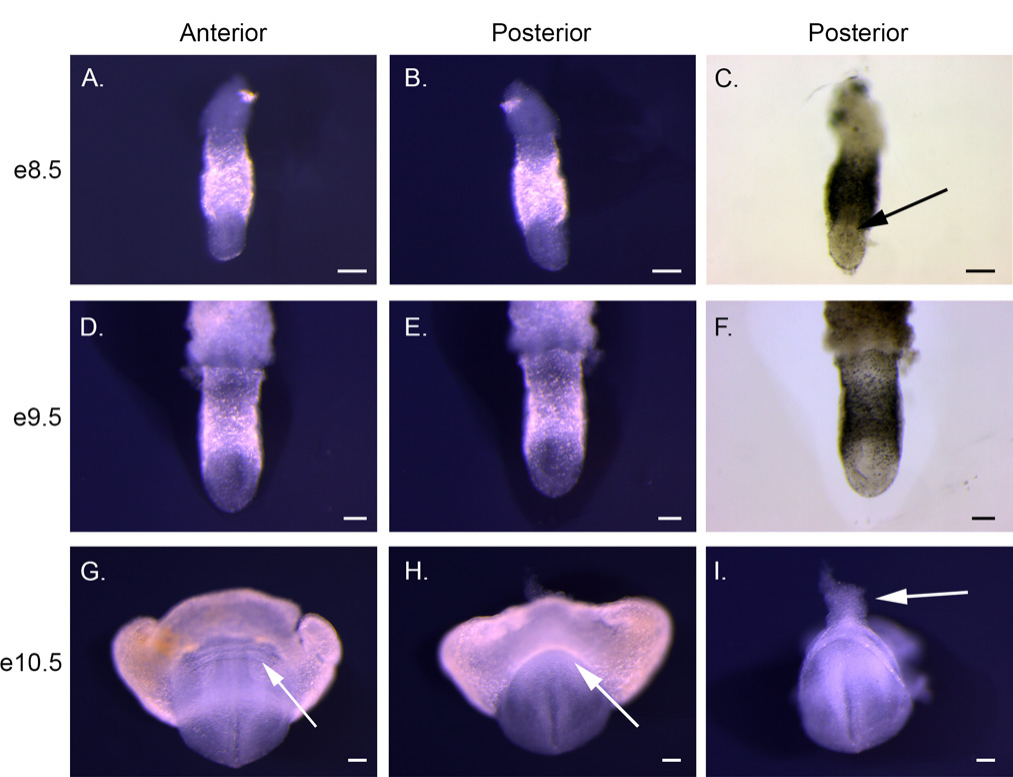
Anterior and posterior views of e8.5, e9.5, and e10.5 *P*. *maniculatus* embryos. (A–C) e8.5 embryo. (D–F) e9.5 embryo. (G–I) e10.5 embryo. (A, D, and G) Anterior view, dark field illumination. Arrow in G indicates the foregut pocket (B, E, and H) Posterior view, dark field illumination. Arrow in H indicates the hindgut pocket (C and F) Posterior view, bright field illumination. Arrow in C indicates the primitive streak. (I) Posterior view, dark field illumination. Extra-embryonic tissues have been removed. Arrow indicates the allantois. All scale bars equal 100 μm.

The morphology of early *P*. *maniculatus* embryos (e8.5) is consistent with the egg cylinder stage of *M*. *musculus* (e6.5) (Fig 1A and Fig 2A–2C). The definitive embryonic tissues are located on the ventral side of the egg cylinder with extra embryonic tissues located dorsally. Asymmetry of the egg cylinder is observed with formation of the primitive streak at e8.5 (Fig 2C). A limiting furrow, characteristic of the anterior of the embryo, forms between the extraembryonic and embryonic tissues at e8.5 (Fig 1A). Thickening of the inner layer of embryonic tissue at e9.5 is consistent with formation of the neural plate (Fig 1B), preceding head fold formation. The primitive streak has lengthened and is no longer distinct (Fig 2F). An open neural plate both anteriorly and posteriorly is apparent at e10.5, with the anterior end having folded ventrally (Fig 1C). Formation of the cardiac crescent is observed below the head fold (Figs 1C, 2G and 3A), and endodermal pockets begin formation at both anterior and posterior ends of the embryo, (Fig 2G–2I). The allantois has formed at the posterior end, and is visible when extra-embryonic tissue is dissected away (Figs 1C and 2I). The first somites also form at e10.5 (Fig 4A).

Embryonic turning occurs in *P*. *maniculatus* between e10.5 and e11.5, and embryos at various stages of turning were observed (data not shown); however, the embryo presented has completed turning at e11.5 (Fig 1D). By e11.5 the basic body plan is complete. The anterior neural tube has closed and regionalization of the anterior neural tube into forebrain, midbrain, and hindbrain is observed. The posterior neural pore remains open. The optic and otic placodes are also visible. Somitogenesis, which initiated on e10.5 (Fig 3A), has progressed at e11.5 as somites are formed from the presomitic mesoderm. The heart tube has begun looping, and the swellings characteristic of the forming atria and ventricles are observed. The pharyngeal arches are developing, with the first arch readily apparent. At e12.5 the forelimb bud is visible (Fig 1E), while a distinct hindlimb bud forms at e13.5 (Fig 1F). From e13.5 to e21.5 embryonic development is characterized by the growth of these structures (Fig 1F–1I and Fig 2A–2E), each of which will be described in greater detail.

### Neural Development

**Fig 5:**
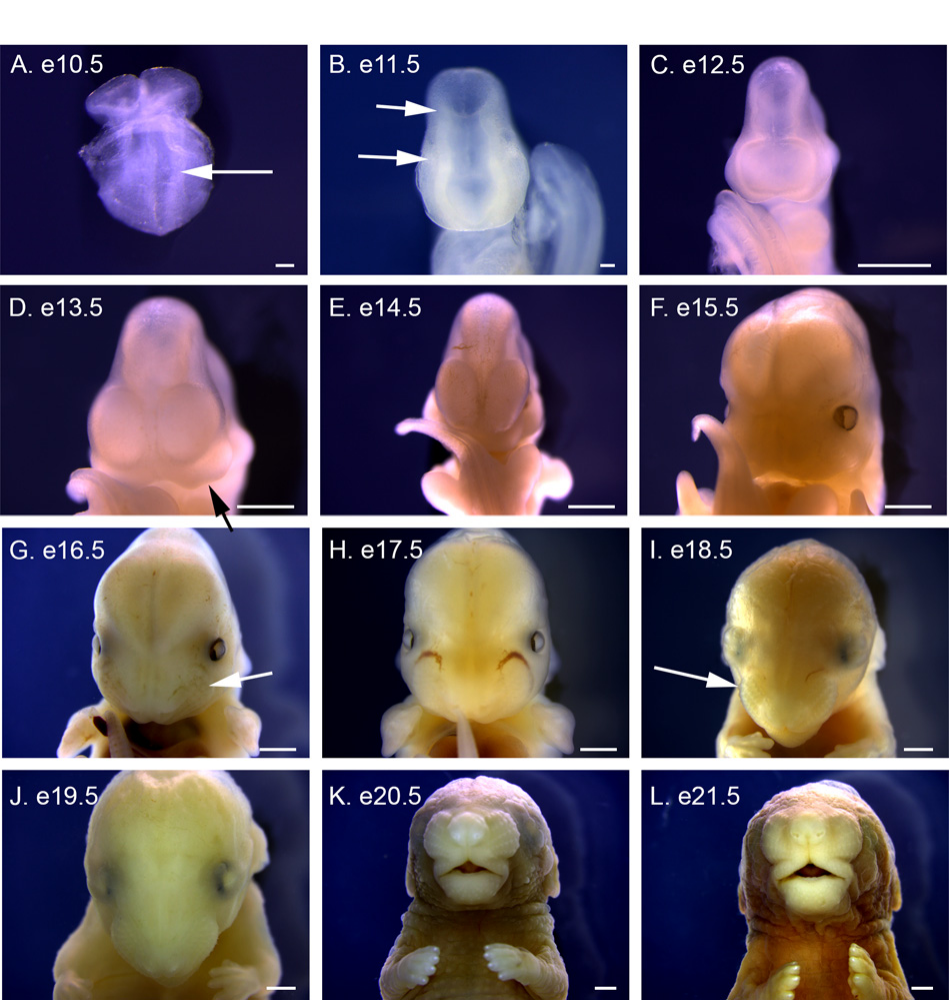
Frontal views of e10.5 to e21.5 *P*. *maniculatus* embryos. (A) e10.5 embryo. Arrow indicates the first pair of somites. (B) e11.5 embryo. Arrows indicate divisions between the telencephalon and diencephalon (lower) and diencephalon and mesencephalon (upper). (C) e12.5 embryo. (D) e13.5 embryo. Arrow indicates olfactory pit. (E) e14.5 embryo. (F) e15.5 embryo. (G) e16.5 embryo. Arrow indicates the prospective area of the forming vibrissae. (H) e17.5 embryo. (I) e18.5 embryo. Arrow indicates the prospective area of the forming vibrissae. (J) e19.5 embryo. (K) e20.5 embryo. (L) e21.5 embryo. Scale bars in A and B equal 100 μm. Scale bars in C–L equal 1 mm.

**Fig 6:**
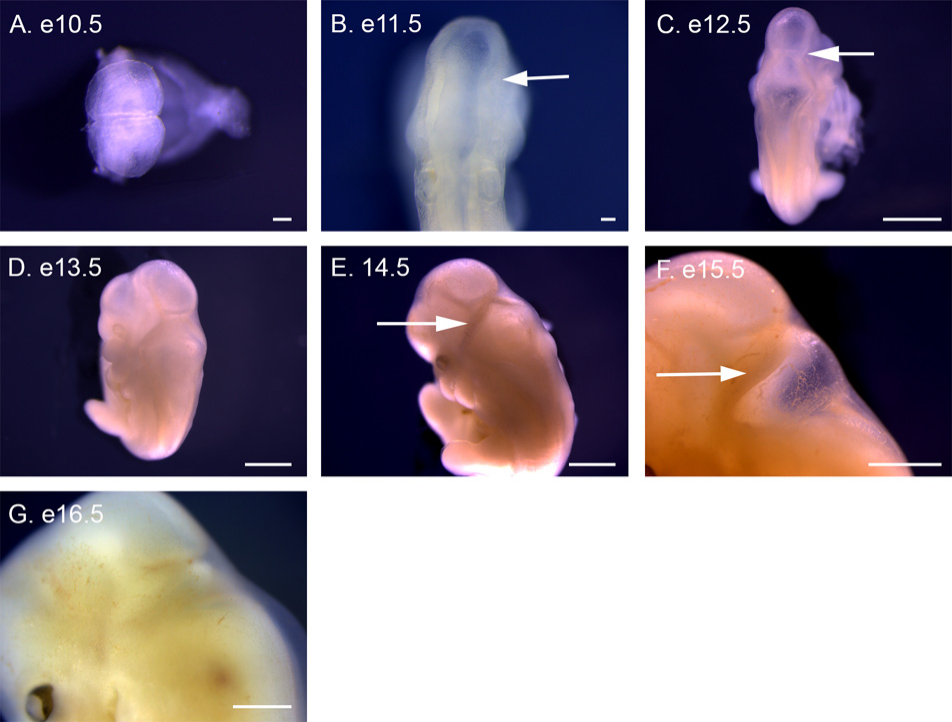
Dorsal views of e10.5 to e16.5 *P*. *maniculatus* embryos. (A) e10.5 embryo. (B) e11.5 embryo. Arrow indicates division between the mesencephalon and rhombencephalon. (C) e12.5 embryo. Arrow indicates division between the mesencephalon and rhombencephalon. (D) e13.5 embryo. (E) e14.5 embryo. Arrow indicates the rhombic lip. (F) e15.5 embryo. Arrow indicates the rhombic lip. (G) e16.5 embryo. Scale bars in A and B equal 100 μm. Scale bars in C–G equal 1 mm.

Frontal images reveal an open neural plate at e10.5 (Fig 5A). One day later, the anterior neural tube has closed and the divisions between the telencephalon, diencephalon, and mesencephalon are apparent (Fig 5B). The paired swellings of the telencephalon are observed at e12.5 (Fig 5C), and they expand significantly from e13.5 to e15.5, forming the cortex (Fig 5D–5F). The formation of the skull, facial structures, and thickening of the skin then obscure observation of the neural tissue at e16.5 (Fig 5G). An open neural plate is also observed in dorsal images at e10.5 (Fig 6A), and the division between mesencephalon and rhombencephalon is apparent at e11.5 (Fig 6B). At e12.5 the rhombencephalon expands forming the characteristic rhombus shape of the hindbrain, and the division with the midbrain is sharpened (Fig 6C). The rhombic lip thickens at e13.5 and is observable through e15.5, before becoming obscured at e16.5 (Fig 6D–6G).

### Eye Development

**Fig 7:**
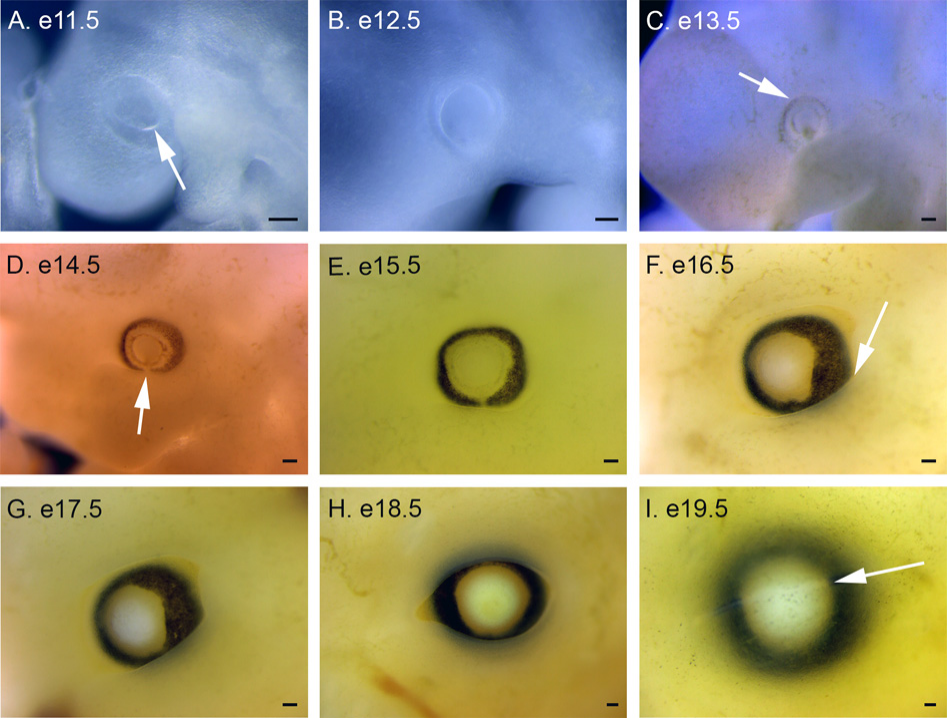
Eye development in *P*. *maniculatus* embryos from e11.5 to e19.5. (A) e11.5 embryo. Arrow indicates the boundary between the optic cup and the invaginating optic placode. (B) e12.5 embryo. (C) e13.5 embryo. Arrow indicates first pigmentation in the RPE. (D) e14.5 embryo. Arrow indicates the choroidal fissure. (E) e15.5 embryo. (F) e16.5 embryo. Arrow indicates the forming eye lid. (G) e17.5 embryo. (H) e18.5 embryo. (I) e19.5 embryo. Arrow indicates the fused eye lids. All scale bars equal 100 μm.

The optic cup is visible at e11.5, encircling the optic placode (Fig 7A). These structures become more refined by e12.5, with the first appearance of the retinal pigmented epithelium (RPE) at e13.5 (Fig 7B & 7C). Pigmentation of the RPE continues at e14.5 and the division between the optic cup and the forming lens is more apparant (Fig 7D). These tissues continue to develop in unison through e15.5, with the lens becoming more opaque at e16.5 (Fig 7E & 7F). The eye lids start to form at e16.5, becoming thicker and narrower at e17.5 and e18.5, before closing over the eye at e19.5 (Fig 7F–7I).

### Ear Development

**Fig 8:**
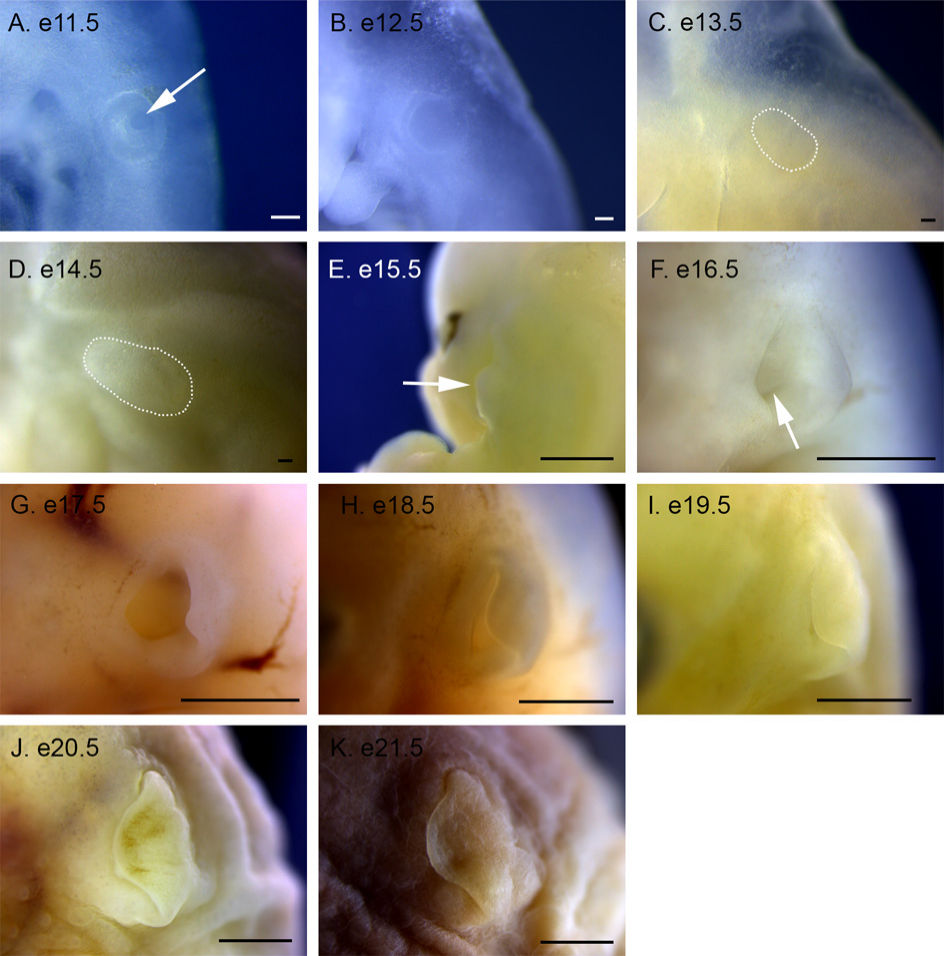
Ear development in *P*. *maniculatus* embryos from e11.5 to e21.5. (A) e11.5 embryo. Arrow indicates invaginating otic placode. (B) e12.5 embryo. (C) e13.5 embryo. Dotted line indicates otic placode, which has become internalized. (D) e14.5 embryo. Dotted line indicates otic placode, which has become internalized. (E) e15.5 embryo, dorsal view. Arrow indicates the pinna. (F) e16.5 embryo. Arrow indicates the auditory meatus. (G) e17.5 embryo. (H) e18.5 embryo. (I) e19.5 embryo. (J) e20.5 embryo. (K) e21.5 embryo. Scale bars in A–D equal 100 μm. Scale bars in E–K equal 1 mm.

The otic placode is visible and has begun invagination by e11.5, and completes invagination by e12.5 (Fig 8A & 8B). The paired placodes are visible adjacent to the hindbrain in dorsal views (Fig 7B & 7C). At e13.5 and e14.5 the otic placode is internalized, and is difficult to observe externally (Fig 8C & 8D). At e15.5 the external ear is first visible as the pinna extends away from the head (Fig 8E). The auditory meatus is visible at e16.5, and the pinna begins to curve rostrally (Fig 8F). The pinna continues to grow, covering more of the auditory meatus from e17.5 to e19.5 until the auditory meatus is completely obscured at e20.5 (Fig 8G–8J). The thickening skin is apparent in the pinna at e21.5 (Fig 8K).

### Pharyngeal arch development

**Fig 9:**
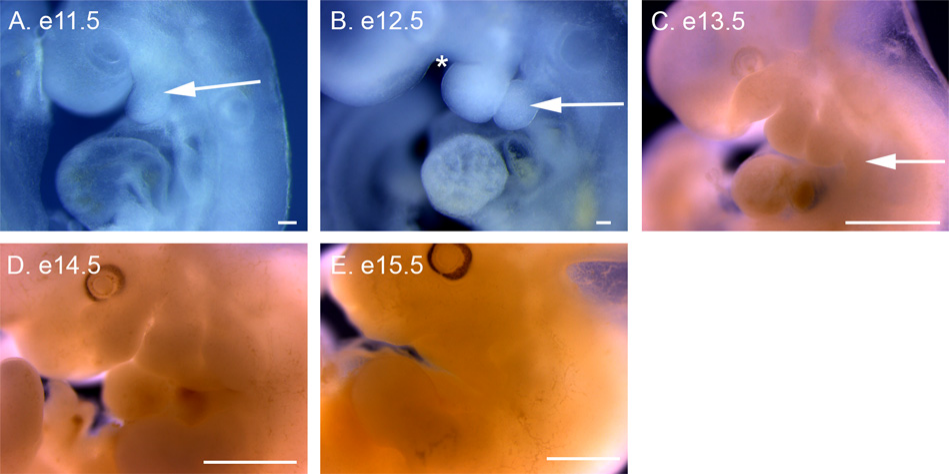
Pharyngeal arch development in *P*. *maniculatus* embryos from e11.5 to e15.5. (A) e11.5 embryo. Arrow indicates the first pharyngeal arch. (B) e12.5 embryo. Arrow indicates the second pharyngeal arch. Asterisk marks the division between maxilla and mandible. (C) e13.5 embryo. Arrow indicates the third pharyngeal arch. (D) e14.5 embryo. (E) e15.5 embryo. Scale bars in A and B equal 100 μm. Scale bars in C–E equal 1 mm.

The first pharyngeal arch is visible at e11.5 (Fig 9A). It continues to grow at e12.5, and a cleft appears separating the first arch from the swelling of the forming maxilla (Fig 9B). The second pharyngeal arch is distinct at e12.5, followed by a swelling indicating the third pharyngeal arch at e13.5 (Fig 9C). At e14.5 the divisions between the arches are becoming obscured and the third pharyngeal arch is no longer distinct (Fig 9D). By e15.5 the pharyngeal arches are no longer apparent, as craniofacial formation progresses and the upper and lower jaws form (Fig 9E).

### Craniofacial development

Lateral views of the pharyngeal arch region highlight the formation of the maxilla and mandible, forming from the first pharyngeal arch, between e12.5 and e15.5 (Fig 9B–9E). Frontal views highlight the forming frontonasal mass. The first indention of the nasal pits occurs at e12.5 and deepen at e13.5 (Fig 5D). The growing frontonasal mass and maxilla narrow the nasal pits to distinguishable nares at e15.5 (Fig 1H). The snout continues to lengthen and rows of vibrissae are visible beginning at e16.5 (Fig 5G), becoming more prominent by e18.5 (Fig 5I). Whisker growth from the vibrissae is visible at e19.5 with lengthening through e21.5 (Fig 5J–5L).

### Limb development

**Fig 10:**
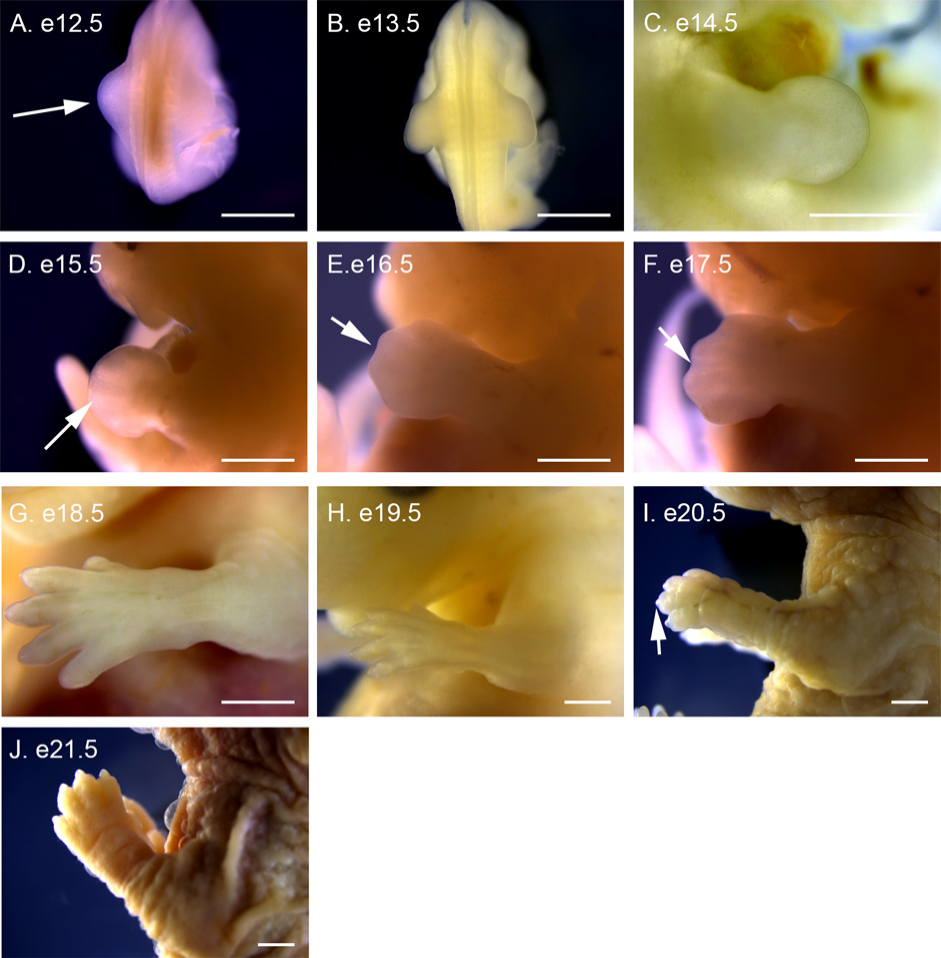
Forelimb development in P. *maniculatus* embryos from e12.5 to e21.5. (A) e12.5 embryo, dorsal view. Arrow indicates left forelimb bud. (B) e13.5 embryo, dorsal view. (C) e14.5 embryo, lateral view of right forelimb. (D–J) Lateral views of left forelimb. (D) e15.5 embryo. Arrow indicates digit condensation. (E) e16.5 embryo. Arrow indicates indentation between digits. (F) e17.5 embryo. Arrow indicates indentation between digits. (G) e18.5 embryo. (H) e19.5 embryo. (I) e20.5 embryo. Arrow indicates forming nail. (J) e21.5 embryo. All scale bars equal 1 mm.

The paired swellings of the forelimb buds extend laterally away from the body at e12.5 (Fig 10A). The forelimb buds become more distinct and angle posteriorly at e13.5 (Fig 10B). A footplate is visible at e14.5 (Fig 10C), with condensations presaging digit formation visible at e15.5 (Fig 10D). The first evidence of digit separation begins at e16.5 with shallow indentations between the digits (Fig 10E), which deepen at e17.5 (Fig 10F), before becoming fully separated at e18.5 (Fig 10G). A distinct bend at the elbow occurs at e17.5 (Fig 10F), and nail formation is apparent at e20.5 (Fig 10H), becoming distinctive at e21.5 (Fig 10I).

**Fig 11:**
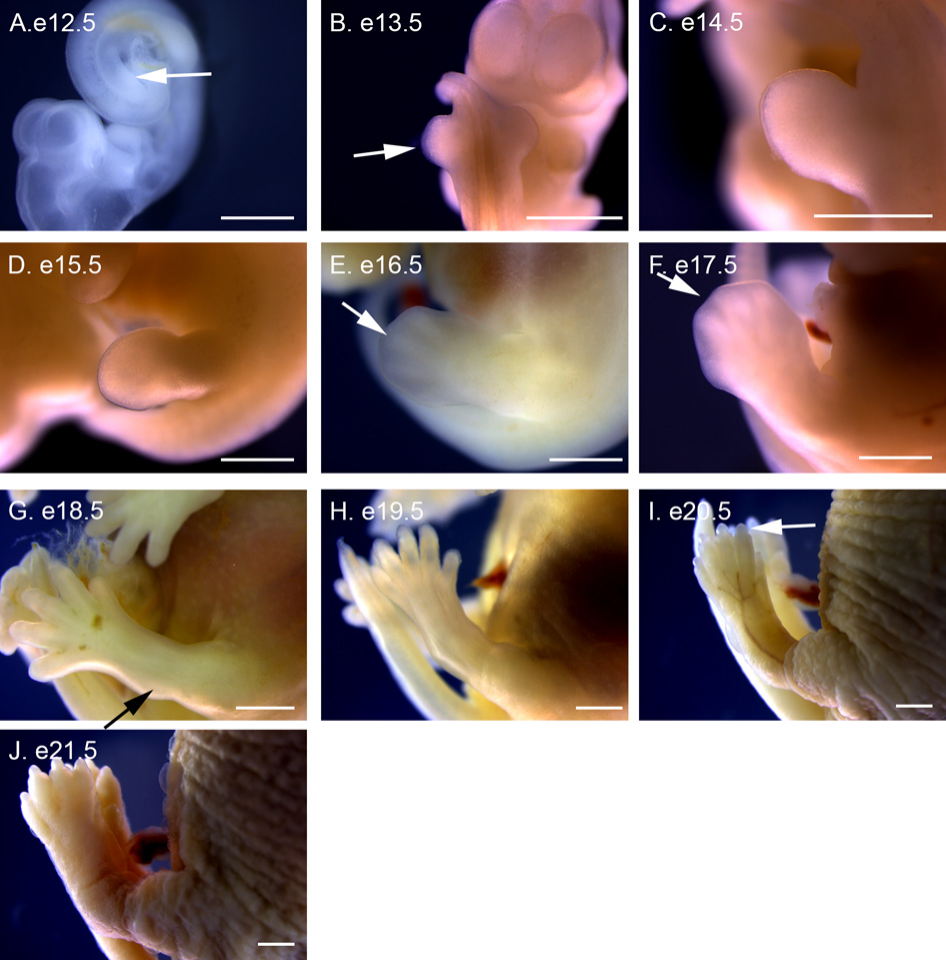
Hindlimb development in P. *maniculatus* embryos from e12.5 to e21.5. (A) e12.5 embryo, lateral view of right side. Arrow indicates right hindlimb bud. (B) e13.5 embryo, dorsal view. Arrow indicates right hindlimb bud. (C–J) Lateral views of left forelimb. (C) e14.5 embryo. (D) e15.5 embryo. (E) e16.5 embryo. Arrow indicates digit condensation. (F) e17.5 embryo. Arrow indicates interdigital tissue. (G) e18.5 embryo. Arrow indicates developing ankle. (H) e19.5 embryo. (I) e20.5 embryo. Arrow indicates forming nail. (J) e21.5 embryo. All scale bars equal 1 mm.

Hindlimb development trails behind forelimb development by roughly one day of development. At e12.5 the first condensations of the hindlimb bud are visible (Fig 11A), becoming distinct limb buds at e13.5 (Fig 11B). The hindlimb bud elongates at e14.5 (Fig 11C), angling anteriorly, and a distinct footplate forms at e15.5 (Fig 11D). Digit condensations are visible at e16.5 (Fig 11E) and digit separation begins at e17.5 (Fig 11F), with full separation by e18.5 (Fig 11G). A swelling at the ankle is visible at e18.5, with a distinct bend forming at e19.5 (Fig 11H). Nails are visible at e20.5 (Fig 11I) and more pronounced at e21.5 (Fig 11J).

## Discussion

The gross morphology of *P*. *maniculatus* development from gastrulation to birth is very similar to the development of *M*. *musculus* [16]. The high degree of similarity between the two suggests that *P*. *maniculatus* researchers can use the numerous resources of *M*. *musculus* development as a general guide for *P*. *maniculatus*, using an appropriate time point correction. Table 1 compares select developmental time points in *M*. *musculus* with *P*. *maniculatus*. Both rodent species have a range of variability in developmental timing for embryos collected on each day of development. Therefore, this table is presented as a guide and not as strict staging criteria. *P*. *maniculatus* trails *M*. *musculus* development by roughly two days at gastrulation, and is caused in part by the increased length of time spent at the 2-cell stage for *P*. *maniculatus* [3]. This two day difference in developmental timing is roughly maintained through limb bud formation. However, the rate of development appears faster in *M*. *musculus*, such that an additional half day of development separates *P*. *maniculatus* and *M*. *musculus* at e14.5 (Mus e12), and an additional full day of development separates the two rodent species at e17.5 (Mus e14.5). Ultimately, a difference of four days separates *P*. *maniculatus* and *M*. *musculus* at birth, which occurs at e24 and e20, respectively. Postnatal development is also slower in *P*. *maniculatus*, as developmental hallmarks such as skin pigmentation, open eyes, weaning, and sexual maturity all occur later than in *M*. *musculus* [3].

**Table 1:** Comparison of Developmental Events in *Peromyscus* and *Mus*.

	Embryonic day of development	
Developmental Event and approximate Mus Theiler stage (TS)	*Mus*	*Peromyscus*
2 to 4-cells (TS 2)	e1.0	e1.5
Blastocyst (TS 5)	e4.0	e6.0
Egg cylinder, embryonic axis visible, primitive streak forms (TS 9–10)	e6.5	e8.5
Neural plate formation (TS 11)	e7.5	e9.5
First somites, cardiac crescent, endodermal pockets (TS 12–13)	e8.5	e10.5
Optic and otic placodes, pharyngeal arches, anterior neural tube closure, heart looping (TS 14–15)	e9.5	e11.5
Distinctive forelimb bud, cleft between maxilla and mandible in first pharyngeal arch (TS 16–17)	e10.5	e12.5
Distinctive hindlimb bud (TS 18–19)	e11.5	e13.5
RPE pigmentation clearly visible (TS 20)	e12	e14.5
Pinna is visible (TS21)	e13	e15.5
Indentions between digits of forelimb bud (TS 22)	e14	e16.5
Indentions between digits of hindlimb bud (TS 22)	e14	e17.5
Separated fingers and toes (TS23)	e15	e18.5
Umbilical hernia absent (TS 24)	e16	e19.5
Wrinkled skin (TS 25)	e17	e20.5
Thick eyelids obscuring eyes (TS26)	e18	e21.5
Birth	e20	e24
Skin pigmentation	P3	P4
Eyes open	P12	P14
Weaning	P20	P24
Sexual maturity	P42	P60
Approximate life span	2 years	4 years

The *Peromyscus* developmental time line presented here will aid the phenotypic characterization of *Peromyscu*s mutants such as *dominant spot*, *tan streak*, and *cataract-webbed*. *Dominant spo*t and *tan streak* are probably caused by defects in neural crest migration, which we can now predict begins at e10.5. The failure to separate the digits, leading to syndactyl in *cataract-webbed*, can now be examined at e16.5 to e17.5 when digit separation occurs in wild type embryos. This initial characterization of *Peromyscus* embryogenesis adds to the growing list of resources available for conducting research on this emerging model system.
